# Amino acid-enriched plant-based RUTF treatment was not inferior to peanut-milk RUTF treatment in restoring plasma amino acid levels among patients with oedematous or non-oedematous malnutrition

**DOI:** 10.1038/s41598-021-91807-x

**Published:** 2021-06-15

**Authors:** Wataru Sato, Chie Furuta, Peter Akomo, Paluku Bahwere, Steve Collins, Kate Sadler, Chrissy Banda, Elizabeth Maganga, Sylvester Kathumba, Hitoshi Murakami

**Affiliations:** 1 Ajinomoto Co. Inc., Research Institute for Bioscience Products & Fine Chemicals, Kawasaki, Japan; 2 Ajinomoto Co. Inc., Institute of Food Science and Technologies, Suzukicho 1-1, Kawasaki-ku, Kawasaki, Kanagawa 210-8681 Japan; 3 Valid Nutrition, Cork, Ireland; 4 Valid International, Oxford, UK; 5 Center for Epidemiology, Biostatistics, and Clinical Research, School of Public Health, Free University of Brussels, Brussels, Belgium; 6 Ministry of Health Malawi, Lilongwe, Malawi

**Keywords:** Health care, Nutrition

## Abstract

Ready-to-use therapeutic food (RUTF) with adequate quality protein is used to treat children with oedematous and non-oedematous severe acute malnutrition (SAM). The plasma amino acid (AA) profile reflects the protein nutritional status; hence, its assessment during SAM treatment is useful in evaluating AA delivery from RUTFs. The objective was to evaluate the plasma AAs during the treatment of oedematous and non-oedematous SAM in community-based management of acute malnutrition (CMAM) using amino acid-enriched plant-based RUTFs with 10% milk (MSMS-RUTF) or without milk (FSMS-RUTF) compared to peanut milk RUTF (PM-RUTF). Plasma AA was measured in a non-blinded, 3-arm, parallel-group, simple randomized controlled trial conducted in Malawi. The RUTFs used for SAM were FSMS-RUTF, MSMS-RUTF or PM-RUTF. A non-inferiority hypothesis was tested to compare plasma AA levels from patients treated with FSMS-RUTF or MSMS-RUTF with those from patients treated with PM-RUTF at discharge. For both types of SAM, FSMS-RUTF and MSMS-RUTF treatments were non-inferior to the PM-RUTF treatment in restoration of the EAA and cystine except that for FSMS-RUTF, methionine and tryptophan partially satisfied the non-inferiority criteria in the oedematous group. Amino-acid-enriched milk-free plant-source-protein RUTF has the potential to restore all the EAA, but it is possible that enrichment with amino acids may require more methionine and tryptophan for oedematous children.

## Introduction

Severe acute malnutrition (SAM) is still a major global issue, with more than 19 million children suffering from SAM in 2011, and SAM accounts for 7.3% of total deaths among children under 5 years of age^1^. SAM is defined by a very low weight for height (below minus 3 z scores of the median WHO growth curves), by the presence of bilateral pitting oedema or by a mid-upper-arm circumference (MUAC) of less than 115 mm in children 6–59 months (mo) of age^2^.

Two clinical types of SAM are recognized: oedematous (kwashiorkor) and non-oedematous (marasmus) malnutrition. Non-oedematous malnutrition is characterized by severe wasting, whereas oedematous malnutrition presents a wide range of clinical signs, including bilateral pitting oedema, loss of hair pigmentation, skin lesions, hypoalbuminemia, and hepatic steatosis. The aetiology of oedematous malnutrition remains a long-time enigma, but this condition responds to dietary treatment, suggesting a key role of some form of macro- or micronutrient deficiency in its aetiology^3^. There are several hypotheses for oedematous malnutrition, such as the possible role of insufficient intake of some amino acids; dysadaptation to a low-protein, high carbohydrate diet; aflatoxins and/or a result of an imbalance between the production of free radicals and their safe disposal^4^; disruption of sulfated glycosaminoglycans; and a possible role of the gut microbiota. However none of these hypotheses have been confirmed^3^. The free radical hypothesis has been supported by several studies; for example, blood concentrations of glutathione (GSH) are lower in children with oedematous malnutrition than in children with non-oedematous malnutrition^5^, and there is excessive lipid peroxidation in oedematous malnutrition (kwashiorkor) as well as excessive quantities of oxidized amino acids^6,7^. However, whether oxidative stress is the primary cause of oedematous malnutrition or one of its many consequences is still unknown^3^. In addition, Giovanni et al. showed that children with oedematous malnutrition (kwashiorkor) were metabolically distinct from those with non-oedematous malnutrition (marasmus) and were more prone to severe metabolic disruptions^8^. According to these data, the 2 types of SAM seem to have different mechanisms. However, due to their effectiveness, ready-to-use therapeutic foods (RUTFs) are used for the treatment of both clinical types of SAM and sometimes in combination with F75 and F100 in cases where SAM treatment starts from the stabilization phase in inpatient facilities^9^.

Currently, regarding the mainstream treatments for SAM, the community-based management of acute malnutrition (CMAM)^10^ approach is used. CMAM approach includes 4 program components; (1) community component for community sensitization and active screening for acute malnutrition, (2) supplementary feeding program component for the management of children with moderate acute malnutrition, (3) outpatient therapeutic program component for the management of children with uncomplicated SAM or those referred from the inpatient component after stabilization, and (4) inpatient care management component of children with complicated SAM. In the outpatient therapeutic component of CMAM, peanut-milk-based RUTF (PM-RUTF) is the most widely used type of nutrition and, combined with early identification of cases and initiation of treatment per CMAM guidelines, is highly effective in the treatment of SAM^10,11^. Efforts have been made to develop alternative RUTF formulations based on plant source proteins using chickpeas, sesame, soya, maize, and sorghum with the aim of replacing the more expensive animal source proteins to reduce product cost by 10 to 25% (rough estimate since prices vary by commodity^12^ and exchange rates, however there is not yet a peer-reviewed published research paper about the cost and cost-effectiveness of these products and further research are expected.) by utilizing the main ingredients available locally in developing countries^13,14^. However, there has been a longstanding controversy regarding the use of plant protein as the sole or major source of protein in RUTFs arising from a concern that the product efficacy may be compromised^15,16^. In an attempt to develop plant protein-based RUTFs intended to be as efficacious as animal protein-based RUTFs, we incorporated the novel concept of amino acid balance in formulating these products and developed RUTFs that are based on locally available ingredients comprising soy, maize, and sorghum (SMS): an amino acid-enriched milk-free RUTF formulation (FSMS-RUTF) and an amino acid-enriched low-milk RUTF formulation containing 9.3% (w/w) milk (MSMS-RUTF). An efficacy trial of the newly developed RUTFs in Malawi showed positive results, suggesting that both are as efficacious as PM-RUTF in the treatment of SAM in terms of recovery rates and are more efficacious in treating anaemia and restoring body iron stores in children aged 6–59 months in treatment arms that all included the same proportions of children diagnosed with oedematous and non-oedematous malnutrition^13,17,18^.

Amino acids are central to metabolism and play key roles in protein and energy metabolism in the body. In particular, EAAs are extremely important for growing children because they are precursors for protein synthesis that must be provided in the diet. Increased plasma EAA concentrations result in increased protein synthesis in human muscles^19^. Moreover, leucine, an EAA, is not only a protein precursor but also a key activating factor for protein synthesis via the mammalian target of rapamycin signalling pathway^20,21^. In addition, there is some evidence that children with oedematous malnutrition (kwashiorkor) have an imbalance between the generation of reactive oxygen species and the available antioxidant capacity, such as in low concentrations of glutathione^4^. The antioxidant GSH is a tripeptide made from glutamate, glycine and cysteine. The major determinant of GSH synthesis is the availability of cysteine- and sulfur-containing amino acid precursors, such as methionine^22^. Thus, amino acids have a wide range of roles in metabolism, hence the importance of measuring plasma amino acid levels to assess the adequacy of bioavailable amino acids during the treatment of SAM.

Since the two SAM conditions described above have important correlations with macro- or micronutrient deficiency, many previous studies have focused on plasma amino acids to clarify the metabolic conditions or mechanisms underlying SAM. In oedematous malnutrition, EAAs excluding phenylalanine, histidine, and threonine and the non-essential amino acids (NEAAs) tyrosine and cysteine tend to be reduced far below normal levels^8,23–25^. In contrast, in non-oedematous malnutrition, the decrease in EAAs is milder than in oedematous malnutrition^8,23^. These results suggest that the metabolism of proteins and/or amino acids is different between patients with oedematous and non-oedematous malnutrition. Therefore, the impact of RUTFs may vary depending on the type of SAM. To investigate this possibility, we compared plasma amino acid profiles during treatment using the three RUTFs for both oedematous and non-oedematous malnutrition to understand the efficacy of each of the two novel RUTFs in treating the two clinical types of SAM.

The study objective was to determine whether treating SAM with an amino acid-enriched plant-based non-milk RUTF (FSMS-RUTF) or with an amino acid-enriched plant-based 10% milk (MSMS-RUTF) is as effective at restoring the plasma concentrations of total EAAs, leucine, methionine and NEAAs, particularly cystine as a GSH precursor, as treatment with the standard milk-based PM-RUTF in oedematous and non-oedematous malnutrition patients in a CMAM programme setting.

## Results

### Baseline characteristics

Table 1 presents the baseline characteristics of the children included in the analyses for each SAM clinical form. In the study, the non-oedematous malnutrition subgroup had a lower weight-for-age z score, height-for-age z score and weight-for-height z score than the oedematous malnutrition subgroup. There were no significant differences (p > 0.05) in any baseline parameters considered in either of the 2 SAM clinical forms between the PM-RUTF arm and the MSMS-RUTF and FSMS-RUTF arms. Breastfeeding rates were not significantly different between the two SAM clinical forms (data not shown).

**Table 1:** **Baseline characteristics of children**.

	Oedematous malnutrition				Non-oedematous malnutrition			
	All	FSMS-RUTF	MSMS-RUTF	PM-RUTF	All	FSMS-RUTF	MSMS-RUTF	PM-RUTF
Participants, n	233	65	98	70	266	89	82	95
Male sex, n	115 (49.4%)	33 (50.8%)	43 (43.9%)	39 (55.7%)	127 (47.7%)	43 (48.3%)	39 (47.6%)	45 (47.4%)
Age, mo	24.8 ± 10.3	23.9 ± 9.2	25.4 ± 10.2	24.7 ± 11.4	16.2 ± 9.0	17.3 ± 9.8	15.6 ± 8.9	15.7 ± 8.2
Weight, kg	8.9 ± 2.1	8.5 ± 2.1	9.2 ± 2.0	8.7 ± 2.4	6.3 ± 1.2	6.3 ± 1.2	6.3 ± 1.3	6.4 ± 1.2
Height, cm	77.0 ± 7.9	75.8 ± 7.1	78.0 ± 7.9	76.6 ± 8.5	68.1 ± 7.0	68.4 ± 7.4	67.9 ± 7.0	68.1 ± 6.6
Midupper arm circumference, mm	126.7 ± 14.0	123.7 ± 13.3	128.5 ± 13.7	127.1 ± 14.8	109.2 ± 5.7	108.8 ± 6.1	109.0 ± 6.2	109.8 ± 4.9
Weight-for-age z score	− 2.6 ± 1.3	− 2.8 ± 1.3	− 2.3 ± 1.2	− 2.7 ± 1.4	− 3.8 ± 1.1	− 4.0 ± 1.1	− 3.8 ± 1.0	− 3.7 ± 1.1
Height-for-age z score	− 2.9 ± 1.4	− 3.2 ± 1.2	− 2.7 ± 1.4	− 3.0 ± 1.4	− 3.7 ± 1.6	− 3.8 ± 1.7	− 3.6 ± 1.4	− 3.6 ± 1.6
Weight-for-height z score	− 1.3 ± 1.4	− 1.5 ± 1.4	− 1.1 ± 1.2	− 1.5 ± 1.4	− 2.5 ± 1.1	− 2.7 ± 1.2	− 2.5 ± 1.0	− 2.4 ± 1.2

Values are expressed as n (%) or means ± SDs unless otherwise indicated.

### Plasma amino acid concentrations

Raw data of the plasma amino acids are shown in Supplementary material (Table S2-S5, Fig S1 A-L). Table 2 presents the predicted plasma amino acid concentrations at admission and discharge in each SAM clinical form.

**Table 2:** **Predicted plasma amino acid concentrations (μM) in each SAM subgroup.**

Amino acids	Oedematous malnutrition						Non-oedematous malnutrition					
	FSMS-RUTF		MSMS-RUTF		PM-RUTF		FSMS-RUTF		MSMS-RUTF		PM-RUTF	
	Admission	Discharge	Admission	Discharge	Admission	Discharge	Admission	Discharge	Admission	Discharge	Admission	Discharge
Methionine	21.3 ± 1.4	23.3 ± 1.6	21.4 ± 1.2	27.8 ± 1.3	20.9 ± 1.3	26.2 ± 1.4	24.0 ± 1.0	24.8 ± 1.2	24.1 ± 1.0	27.0 ± 1.2	24.2 ± 0.9	25.1 ± 1.1
Leucine	102.6 ± 4.9	129.8 ± 5.8	97.4 ± 4.1	134.1 ± 4.7	102.6 ± 4.8	121.4 ± 5.3	118.8 ± 4.1	135.3 ± 5.3	119.6 ± 4.1	144.8 ± 5.0	121.8 ± 3.9	120.6 ± 4.7
Valine	156.6 ± 7.9	194.9 ± 9.4	148.9 ± 6.6	204.9 ± 7.6	157.4 ± 7.7	207.4 ± 8.5	187.0 ± 7.0	204.0 ± 9.0	186.2 ± 7.1	229.9 ± 8.5	194.0 ± 6.7	196.4 ± 8.1
Isoleucine	70.4 ± 3.6	77.7 ± 4.2	64.4 ± 3.0	81.5 ± 3.4	66.0 ± 3.4	76.2 ± 3.7	79.4 ± 3.0	79.3 ± 3.9	78.1 ± 3.1	89.3 ± 3.7	78.5 ± 2.9	73.5 ± 3.5
Lysine	171.9 ± 9.8	208.1 ± 11.5	165.3 ± 8.2	221.0 ± 9.3	156.5 ± 9.6	194.6 ± 10.4	201.5 ± 8.1	185.9 ± 10.4	197.2 ± 8.2	225.8 ± 9.8	188.4 ± 7.8	182.6 ± 9.4
Phenylalanine	73.2 ± 3.1	74.5 ± 3.7	72.0 ± 2.6	79.5 ± 3.0	72.4 ± 3.1	83.7 ± 3.3	78.6 ± 2.4	70.3 ± 3.1	74.6 ± 2.4	82.5 ± 2.9	76.6 ± 2.2	71.9 ± 2.7
Tryptophan	17.0 ± 1.5	24.6 ± 1.7	15.7 ± 1.2	28.6 ± 1.4	16.1 ± 1.5	29.0 ± 1.6	26.3 ± 1.2	28.3 ± 1.5	25.6 ± 1.2	32.5 ± 1.5	26.9 ± 1.1	28.2 ± 1.4
Threonine	77.4 ± 3.7	86.9 ± 4.4	76.2 ± 3.1	81.1 ± 3.6	80.1 ± 3.6	89.6 ± 4.0	92.9 ± 3.4	88.0 ± 4.4	90.2 ± 3.5	101.1 ± 4.2	96.4 ± 3.3	93.3 ± 4.0
Histidine	70.4 ± 2.3	71.6 ± 2.7	70.4 ± 1.9	74.6 ± 2.2	71.4 ± 2.3	72.3 ± 2.5	73.7 ± 1.7	74.4 ± 2.2	72.6 ± 1.7	74.7 ± 2.1	75.1 ± 1.6	69.9 ± 2.0
Total BCAA	329.6 ± 15.9	402.5 ± 18.9	310.7 ± 13.3	420.4 ± 15.2	325.7 ± 15.5	404.7 ± 16.9	385.2 ± 13.8	418.7 ± 17.7	383.9 ± 13.9	464.1 ± 16.8	394.2 ± 13.2	390.5 ± 16.0
Total EAA	810.0 ± 37.1	890.3 ± 42.1	768.7 ± 30.9	967.4 ± 35.0	778.3 ± 35.3	920.1 ± 39.6	882.8 ± 26.4	913.6 ± 34.0	872.6 ± 26.6	1000.2 ± 32.7	884.4 ± 25.1	856.9 ± 30.4
Cystine	19.0 ± 0.8	27.0 ± 0.9	18.9 ± 0.7	25.7 ± 0.8	20.1 ± 0.8	29.5 ± 0.9	24.4 ± 0.7	29.0 ± 0.9	24.0 ± 0.7	30.6 ± 0.8	25.4 ± 0.7	29.8 ± 0.8

Plasma amino acid concentrations were predicted from a mixed model with a random intercept by using oedematous and non-oedematous malnutrition subgroup data. Fixed effects were intervention (RUTFs) and sampling point (admission or discharge). The model was adjusted for the admission values and random effects of the sub-administrative areas. Predicted concentrations are expressed as the means ± SEs (μM).

All specimens described as “Analysis” in Fig. 2 were used for this analysis.

### Noninferiority analyses of plasma amino acids in oedematous and non-oedematous malnutrition

**Figure 1:**
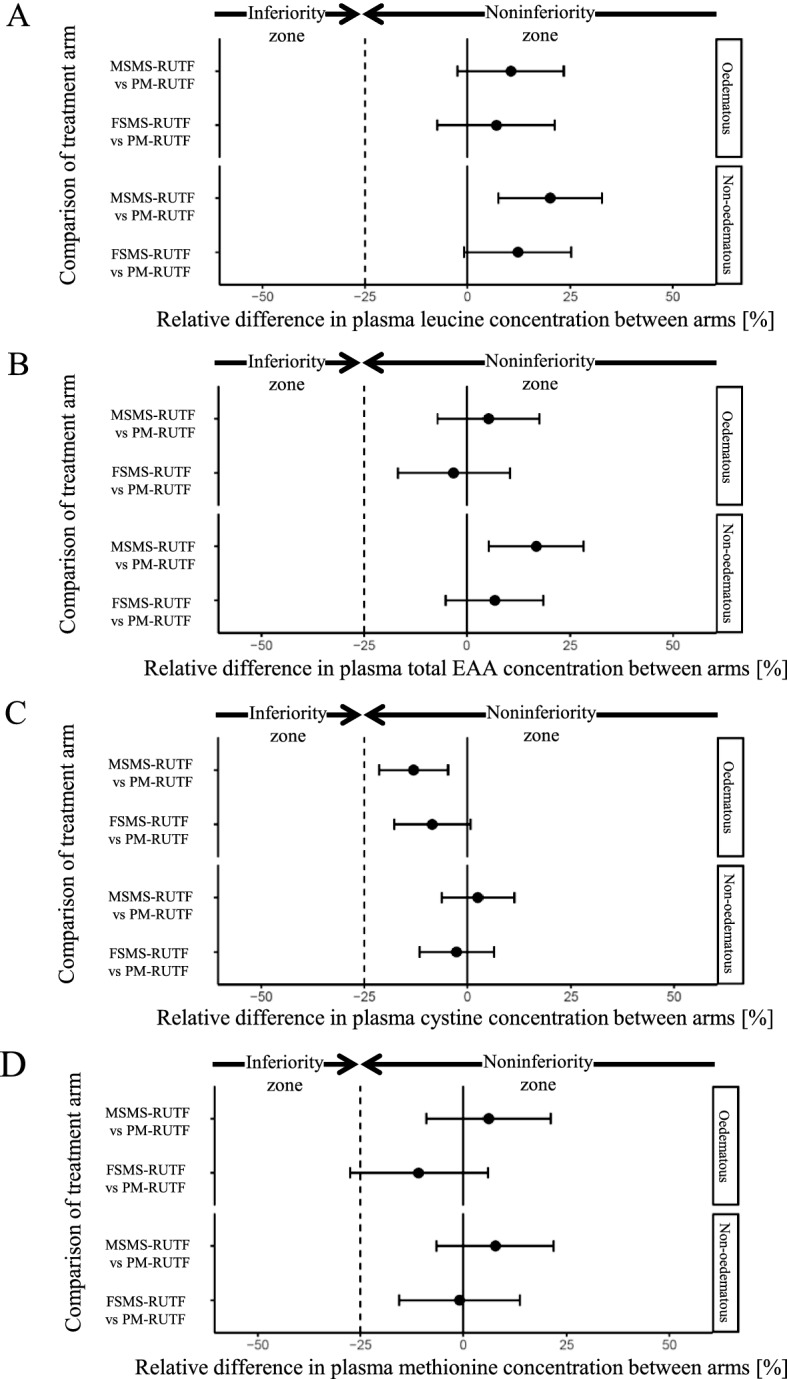
**Comparison of the difference in plasma amino acid concentrations at discharge between the FSMS-RUTF arm and PM-RUTF arm and between the MSMS-RUTF arm and PM-RUTF arm by using the data from each SAM subgroup**. The differences are shown as the relative value based on the plasma amino acid concentration of the PM-RUTF arm at discharge; 0% for the x-axis indicates the mean concentration of the PM-RUTF arm at discharge; 95% CIs were estimated by simultaneous inference procedures in the mixed model. The filled circle and error bar indicate a point estimate of the difference and 95% CI. The dotted line shows the noninferiority margin (− 25%). All specimens described as “Analysis” in Fig. 2 were used for this analysis. **(A)** Relative difference in plasma leucine concentration between arms. **(B)** Relative difference in plasma total EAA concentration between arms. **(C)** Relative difference in plasma cystine concentration between arms. **(D)** Relative difference in plasma methionine concentration between arms.

In oedematous malnutrition, the relative values of the difference (95% CI) in plasma leucine concentration based on the PM-RUTF arm at discharge were 6.9% (− 7.3, + 21.2) and 10.5% (− 2.4, + 23.4) for the FSMS-RUTF and MSMS-RUTF arms, respectively (Fig. 1A, Table 3). In non-oedematous malnutrition, the relative values of the difference (95% CI) in the leucine concentration based on the PM-RUTF arm at discharge were 12.2% (− 0.8, + 25.2) and 20.1% (+ 7.4, + 32.7) for the FSMS-RUTF and MSMS-RUTF arms, respectively (Table 3). Actual values are shown in the Supplementary data (Table S1). These results indicate that the plasma leucine concentrations of the FSMS-RUTF and MSMS-RUTF arms were non-inferior to those of the PM-RUTF arm at discharge in both SAM clinical forms.

**Table 3:** **Testing the non-inferiority of the plasma amino acid concentrations at discharge between the PM-RUTF arm and the FSMS-RUTF and MSMS-RUTF arms in each SAM subgroup.**

Amino acids	Comparison of treatment arm	Oedematous malnutrition		Non-oedematous malnutrition	
		Difference (%)	(95% CI)^1^	Difference (%)	(95% CI)^1^
Methionine	FSMS-RUTF vs PM-RUTF	− 10.9	(− 27.6, 5.9)	− 0.9	(− 15.6, 13.7)
	MSMS-RUTF vs PM-RUTF	6.1	(− 9.0, 21.1)	7.7	(− 6.5, 21.8)
Leucine	FSMS-RUTF vs PM-RUTF	6.9	(− 7.3, 21.2)	12.2	(− 0.8, 25.2)
	MSMS-RUTF vs PM-RUTF	10.5	(− 2.4, 23.4)	20.1	(7.4, 32.7)
Valine	FSMS-RUTF vs PM-RUTF	− 6.0	(− 19.4, 7.4)	3.9	(− 9.8, 17.6)
	MSMS-RUTF vs PM-RUTF	− 1.2	(− 13.3, 10.9)	17.1	(3.7, 30.3)
Isoleucine	FSMS-RUTF vs PM-RUTF	1.9	(− 14.3, 18.1)	7.9	(− 7.9, 23.7)
	MSMS-RUTF vs PM-RUTF	6.9	(− 7.6, 21.4)	21.6	(6.2, 36.9)
Lysine	FSMS-RUTF vs PM-RUTF	6.9	(− 10.6, 24.5)	1.8	(− 15.2, 18.8)
	MSMS-RUTF vs PM-RUTF	13.5	(− 2.1, 29.2)	23.7	(7.2, 40.1)
Phenylalanine	FSMS-RUTF vs PM-RUTF	− 11.0	(− 24.2, 2.2)	− 2.1	(− 14.8, 10.5)
	MSMS-RUTF vs PM-RUTF	− 5.0	(− 16.8, 6.8)	14.8	(2.6, 27.0)
Tryptophan	FSMS-RUTF vs PM-RUTF	− 15.4	(− 33.5, 2.7)	0.3	(− 15.5, 16.0)
	MSMS-RUTF vs PM-RUTF	− 1.6	(− 18.0, 14.8)	15.2	(− 0.4, 30.7)
Threonine	FSMS-RUTF vs PM-RUTF	− 3.0	(− 17.8, 11.7)	− 5.7	(− 19.9, 8.5)
	MSMS-RUTF vs PM-RUTF	-9.5	(− 22.8, 3.8)	8.4	(− 5.5, 22.2)
Histidine	FSMS-RUTF vs PM-RUTF	− 1.0	(− 12.2, 10.3)	6.4	(− 2.9, 15.7)
	MSMS-RUTF vs PM-RUTF	3.1	(− 7.0 , 13.2)	6.8	(− 2.2, 15.9)
Total BCAA	FSMS-RUTF vs PM-RUTF	− 0.6	(− 14.4, 13.3)	7.2	(− 6.3, 20.8)
	MSMS-RUTF vs PM-RUTF	3.9	(− 8.6, 16.4)	18.8	(5.7, 32.0)
Total EAA	FSMS-RUTF vs PM-RUTF	− 3.2	(− 16.8, 10.3)	6.6	(− 5.2, 18.4)
	MSMS-RUTF vs PM-RUTF	5.1	(− 7.2 , 17.5)	16.7	(5.2, 28.2)
Cystine	FSMS-RUTF vs PM-RUTF	− 8.5	(− 17.7, 0.8)	− 2.6	(− 11.6, 6.3)
	MSMS-RUTF vs PM-RUTF	− 13.0	(− 21.4, − 4.7)	2.5	(− 6.2, 11.3)

The point estimate and 95% CI of the differences in plasma amino acid concentrations at discharge between the FSMS-RUTF and PM-RUTF arms and between the MSMS-RUTF and PM-RUTF arms by using the SAM subgroup data. The differences are shown as relative values based on the plasma amino acid concentrations of the PM-RUTF arm at discharge.

^1^CIs were estimated by simultaneous inference procedures in a mixed model. The non-inferiority margin was -25% of the plasma amino acid concentration of the PM-RUTF arm at discharge. Therefore, the lower limit of 95% CI above -25% indicated non-inferiority.

**Figure 2:**
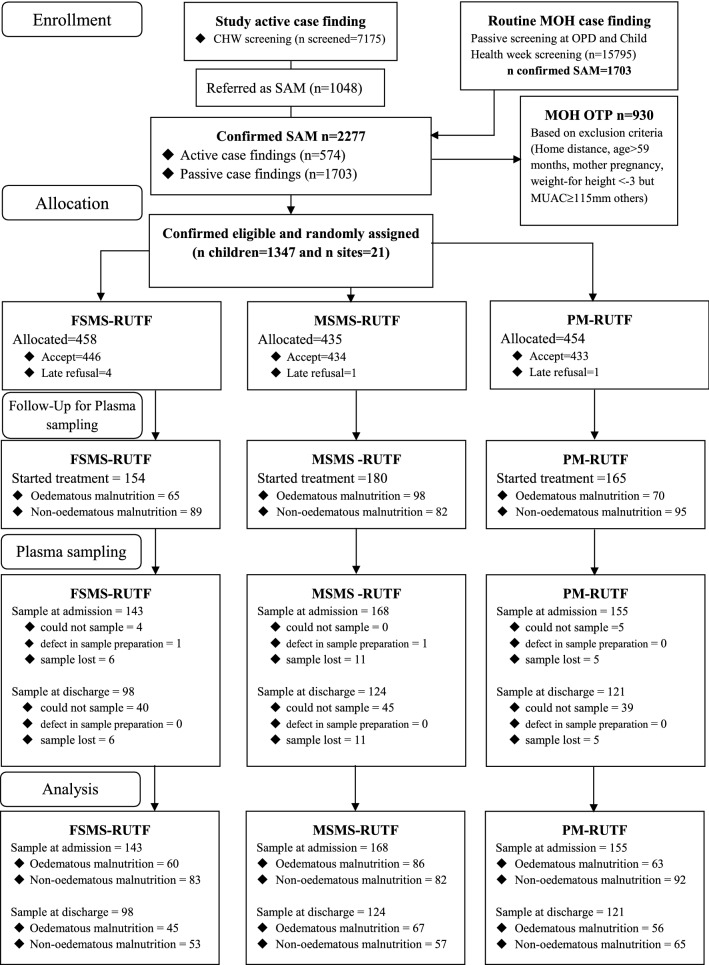
**Study participant flow diagram**. *CHW* community health worker, *MOH* Ministry of Health, *OPD* outpatient department, *OTP* outpatient programme, *SAM* severe acute malnutrition, *MUAC* mid-upper arm circumference, *FSMS* milk-free, soya, maize, and sorghum, *RUTF* ready-to-use therapeutic food, *MSMS* milk, soya, maize, and sorghum, *PM* peanut and milk.

All specimens described as “Analysis” in Fig. 2 were used for this analysis.

Similarly, the plasma concentrations of total EAAs and cystine in the FSMS-RUTF and MSMS-RUTF arms were also non-inferior to the concentrations in the PM-RUTF arm in both SAM clinical forms (Fig. 1B,C, Table 3). Plasma cystine level was significantly lower in the MSMS-RUTF oedematous SAM compared to the PM-RUTF oedematous SAM (Fig. 1C).

For oedematous malnutrition, the relative values of the difference (95% CI) in plasma methionine concentration based on the PM-RUTF arm at discharge were − 10.9% (− 27.6, + 5.9) and 6.1% (− 9.0, + 21.1) for the FSMS-RUTF and MSMS-RUTF arms, respectively (Fig. 1D, Table 3). In non-oedematous malnutrition, the relative values of the difference (95% CI) in the methionine concentration based on the PM-RUTF arm at discharge were − 0.9% (− 15.6, + 13.7) and 7.7% (− 6.5, + 21.8) for the FSMS-RUTF and MSMS-RUTF arms, respectively (Table 3). Actual values are shown in the Supplementary material (Table S1). These results indicated that the plasma methionine concentrations in the FSMS-RUTF arm for non-oedematous malnutrition and the MSMS-RUTF arm for both SAM clinical forms were non-inferior to those in the PM-RUTF arm at discharge.

### Plasma amino acids in oedematous and non-oedematous malnutrition

Table 4 presents the predicted plasma amino acid concentrations at admission and discharge in each SAM clinical form. For the oedematous malnutrition subgroup, methionine, leucine, valine, isoleucine, lysine, tryptophan, total BCAAs, total EAAs and cystine were predicted to be significantly higher at discharge than at admission. For the non-oedematous malnutrition subgroup, leucine, valine, tryptophan, total BCAAs and cystine were significantly higher at discharge than at admission.

**Table 4:** **Predicted plasma amino acid concentrations and comparison between admission and discharge for each SAM subgroup.**

Amino acids	Oedematous malnutrition				Non-oedematous malnutrition			
	Admission	Discharge	p-value^1^		Admission	Discharge	p-value^1^	
Methionine	21.7 ± 1.3	26.2 ± 1.3	<0.001	***	24.0 ± 1.2	25.6 ± 1.3	0.263	
Leucine	103.6 ± 4.8	131.9 ± 5.1	<0.001	***	120.0 ± 4.4	133.7 ± 4.9	0.008	**
Valine	158.3 ± 7.2	208.4 ± 7.7	<0.001	***	189.5 ± 6.5	211.0 ± 7.4	0.009	**
Isoleucine	68.1 ± 3.4	80.8 ± 3.6	<0.001	***	78.3 ± 3.1	80.7 ± 3.5	0.935	
Lysine	171.6 ± 11.5	215.0 ± 11.9	<0.001	***	194.5 ± 10.8	198.0 ± 11.6	1.000	
Phenylalanine	73.2 ± 2.4	78.3 ± 2.6	0.200		76.6 ± 2.1	75.3 ± 2.5	1.000	
Tryptophan	16.5 ± 1.3	27.6 ± 1.4	<0.001	***	26.4 ± 1.2	29.5 ± 1.3	0.037	*
Threonine	79.9 ± 3.4	87.8 ± 3.7	0.086		93.5 ± 3.1	94.0 ± 3.6	1.000	
Histidine	74.8 ± 2.7	77.1 ± 2.8	0.560		73.9 ± 2.5	73.7 ± 2.7	1.000	
Total BCAA	330.2 ± 15.2	421.4 ± 16.1	<0.001	***	387.8 ± 13.8	425.5 ± 15.6	0.026	*
Total EAA	809.5 ± 35.7	943.1 ± 36.5	<0.001	***	881.6 ± 32.4	930.2 ± 35.3	0.210	
Cystine	19.7 ± 0.9	27.8 ± 0.9	<0.001	***	24.7 ± 0.8	29.8 ± 0.9	<0.001	***

Predicted concentrations are expressed as the means ± SEs (μM).

^1^P-values were obtained from the general linear hypotheses test (null hypothesis: the difference between admission and discharge = 0), and p < 0.05 indicated a significant difference between admission and discharge.

All specimens described as “Analysis” in Fig. 2 were used for this analysis.

## Discussion

### Non-inferiority of the newly developed RUTFs compared to PM-RUTF in restoring plasma amino acid concentrations

The objective of this study was to evaluate whether treatment with FSMS or MSMS-RUTF could restore total EAA (sum of methionine, leucine, valine, isoleucine, lysine, phenylalanine, tryptophan, threonine and histidine), leucine, methionine and cystine to levels comparable to those observed after treatment with PM-RUTF among children with SAM (oedematous or non-oedematous malnutrition). The present study demonstrated the non-inferiority of the newly developed RUTFs compared to PM-RUTF with regard to restoring the concentrations of total EAAs, each EAA and cystine in children with non-oedematous malnutrition. In children with oedematous malnutrition, the plant-based amino acid-enriched recipes were non-inferior in restoring total EAAs, cystine and each EAA, with the exception of methionine and tryptophan. Regarding the use of FSMS-RUTF for the treatment of oedematous malnutrition, the results of two plasma amino acids, methionine and tryptophan, were inconclusive, which means that the 95% CI was below the − 25% non-inferiority margin. This does not mean that the FSMS-RUTF is inferior to PM-RUTF in the treatment of oedematous malnutrition since the point estimation did not drop below − 25% (Table 3). A larger sample size may be required to confirm non-inferiority, but this treatment is very likely non-inferior since at least the point estimate is not below the non-inferiority margin. These results confirm the non-inferiority of the treatments in terms of total EAA, leucine and cystine concentrations, and the point estimate of methionine suggests that the restoration of the concentrations of these amino acids was one of the key factors supporting the efficacy of the two novel formulations for the treatment of both oedematous and non-oedematous malnutrition.

Based on previous research, we proposed that restoring plasma leucine and cystine levels was essential for the recovery of SAM^17^. Leucine is not only a precursor for protein synthesis but also a key activating factor for protein synthesis via the mammalian target of rapamycin signalling pathway^20,21^. Therefore, if nutritional therapy is given to SAM patients, achieving adequate levels of plasma leucine may change metabolism from proteolysis to protein synthesis. In addition, cystine has an important role in recovery from oedematous malnutrition as a GSH precursor^26,27^. In children with oedematous malnutrition, the GSH concentration in the blood is lower than that in healthy subjects^4,5,28^, indicating that oxidative stress plays an important role in the pathophysiology of oedematous malnutrition and that recovery of plasma cystine, which is a precursor for GSH, is important for treatment of oedematous malnutrition. Of note, although non-inferiority was confirmed in the FSMS-RUTF and MSMS-RUTF arm vs the PM-RUTF, a significantly lower plasma cystine level was detected in the MSMS-RUTF oedematous malnutrition group than in the PM-RUTF oedematous malnutrition group (Table 3). Since methionine is a cysteine precursor, the high demand for cysteine for GSH synthesis might have resulted in the lower plasma sulfur amino acids in the FSMS-RUTF and MSMS-RUTF groups compared to the PM-RUTF group, possibly due to various reasons such as digestibility of the plant proteins. The formulation of the present developed RUTFs has shown non-inferiority in terms of clinical outcomes such as recovery rate and program length of stay^13^. The analysis of the previous and present studies demonstrates that adding more methionine and cysteine as a source of sulfur amino acids can be an effective way to ensure beneficial changes to the plasma amino acid concentrations^29^. Compared to the SMS-RUTF prototype used in the DRC study, which was a cereal-based RUTF without crystal amino acids^30^, patients treated with the newly developed FSMS- and MSMS-RUTF were able to achieve higher plasma amino acid concentrations at discharge, suggesting the improved efficacy of the products after enhancement with amino acids. The only amino acid that differed from those in the DRC study^30^, assuming the plasma cystine concentration at discharge for the PM-RUTF arm was 100%, was the cystine concentration, which dropped to 70% in the prototype-RUTF arm in the previous study. In contrast, in the present study, the cystine concentration for the FSMS-RUTF arm was 92% and 97% in the oedematous and non-oedematous malnutrition subgroups, respectively (Fig. 1C, Table 3). Since the study environments, baseline characteristics of the subjects, number of subjects analysed and subgroup analyses are different between the two studies, we cannot draw any causal conclusions. However, the restoration of plasma amino acids likely contributed to the improvement in recovery rates compared with non-amino acid-enhanced SMS RUTF. Further research is needed to form definitive conclusions regarding this observation.

The objective of this study was to evaluate whether FSMS or MSMS-RUTF treatment could restore amino acids to levels comparable to those in children treated with PM-RUTF in both types of SAM. As a premise of this evaluation, we confirmed that FSMS and MSMS-RUTFs were as effective as the standard PM-RUTF for promoting nutritional recovery in both children admitted with oedematous SAM and in those admitted for non-oedematous SAM according to the same data as shown in our previous publication and in supplementary table S6^13^. These findings indicate that the newly developed RUTFs can be used in countries with a predominance of oedematous SAM as well as countries with a predominance of non-oedematous SAM. In addition, promoting nutritional recovery suggests that it may be associated with recovery of plasma amino acid levels.

One important limitation in this present study is that upon the blood draw, we could not control the mealtime of the subjects due to ethical reasons. Plasma amino acid concentrations fluctuate after protein from breastmilk and meals. To detect stable baseline levels of plasma amino acids, the timing of the blood draw would be ideally after a sufficient time interval of the last intake. However, many of the study subjects were severely malnourished young children and were still breastfed and required frequent feeding; we prioritized the safety of the children and did not enforce fasting upon the blood draw. Therefore, the background of the subjects might theoretically limit the generalizability of the present study. Nonetheless, there were no significant differences in the breastfeeding rate between the treatment arms, illustrating that the conditions between the different arms were equal. Therefore, the results may be generalized even in a study population in which breastfed children are included^17^. Another limitation of the study was that normal levels of plasma amino acids of children are not defined. For this reason, we could not accurately conclude which plasma amino acid concentrations were restored to the normal level and which amino acid is most important for recovery from SAM. However, we could evaluate how the FSMS or MSMS RUTFs we developed affected plasma amino acids compared to the PM-RUTF as an active control.

### Plasma amino acid concentrations in oedematous and non-oedematous malnutrition

The changes in each EAA in the oedematous and non-oedematous malnutrition groups are shown in Table 4. In the oedematous malnutrition group, the plasma concentrations of almost all EAAs, with the exception of phenylalanine, threonine and histidine, were significantly lower at admission than at discharge. The only EAAs that were significantly lower in the non-oedematous malnutrition group than in the oedematous malnutrition group were leucine, valine and tryptophan. Additionally, for cystine, which is an NEAA, both the oedematous and non-oedematous malnutrition groups showed significantly lower levels at admission than at discharge. These results were consistent with those of previous studies^8,23–25^.

In previous studies and similar to our current results, the plasma concentrations of leucine, valine, tryptophan and cystine were found to be lower in both the oedematous and non-oedematous malnutrition groups than their concentrations at discharge. The turnover of muscle protein and oxidation of leucine are suppressed in children with malnutrition, particularly in those showing oedema^31–34^. Our data show that both subgroups had significantly low concentrations of plasma amino acids leucine and valine at admission compared to discharge. However, the concentrations in the oedematous malnutrition group were significantly lower than those in the non-oedematous malnutrition at admission, which was very similar to the data from previous studies.

Three amino acids—methionine, isoleucine and lysine—were distinct in the oedematous malnutrition group compared to the non-oedematous malnutrition group at admission. Giovanni et al.^8^ found that lysine and methionine are at lower concentrations in the plasma of children with oedematous malnutrition (kwashiorkor) than the plasma of those with non-oedematous malnutrition (marasmus). Lysine and methionine are required for the NAD-dependent biosynthesis of carnitine. Carnitine is essential for the transfer of long-chain fatty acids across the inner mitochondrial membrane for subsequent β-oxidation. Several studies show that children with oedematous malnutrition (kwashiorkor) have lower concentrations of acylcarnitines, which are markers of β-oxidation^8,35^, and children with oedematous malnutrition (kwashiorkor) are known to have high concentrations of plasma fatty acids^36^. It is not clear whether the low levels of lysine and methionine in children with oedematous malnutrition are a cause or effect, but these amino acids may provide a link to the disorder of lipid metabolism seen in these children.

### Protein source in RUTFs

There is controversy over the efficacy of RUTFs with regard to the source of protein used (milk versus plant) for the treatment of protein and energy malnutrition, as milk source-based formulations are believed to be more efficacious^16^. The findings from previous studies and the present study demonstrate that the efficacy of plant-based RUTF is achievable by enhancing the quality of protein and amino acid balance through enrichment of crystallized amino acids. This conclusion is supported by the non-inferiority in recovery rates and of most plasma essential amino acids (excluding methionine and tryptophan) after discharge in the FSMS and MSMS-RUTF treatment patients in relation to those treated with PM-RUTF, which is the major product used globally to treat SAM patients. The concept of calculating the amino acid balance might have been the key to solving the long-term controversy surrounding this issue. The present research has clarified in detail how treatment of SAM using the three amino RUTFs impacts plasma amino acids in children and provides a method to improve the quality of plant-based RUTFs. These plant-based RUTFs, which are also more efficacious in treating anaemia and restoring body iron stores^18^, are less expensive and easier to produce in developing countries, which can increase access to these life-saving products.

## Conclusion

For individuals suffering from oedematous and non-oedematous malnutrition, restoration of the plasma cystine and essential amino acid concentrations at recovery, except for methionine and tryptophan, which were inconclusive, did not differ according to the types of RUTF used for treatment in the present CMAM programme setting.

## Methods

### Study design

This study was part of a nonblinded, 3-arm, parallel-group, simple randomized controlled trial conducted in the central region of Malawi. The trial examined the efficacy of FSMS-RUTF (0% milk powder), MSMS-RUTF (9.3% milk powder) and PM-RUTF (25% milk powder) in the treatment of SAM among children aged 6–59 mo. This trial was registered at the Pan African Clinical Trials Registry as trial no. PACTR201505001101224 on April 15, 2015.

### Ethics

Before data collection began, we obtained permission to conduct the study from the National Ethics Committee of the Malawi Ministry of Health and from the Ajinomoto Institutional Review Board. At the time of admission, each child’s parent or caregiver was informed about the nature of the study and provided informed consent for their child to be included and for their child’s medical information to be used for research purposes. Given the short duration of the study, no interim analysis was planned, and no stopping rule was predefined. No serious side effects were detected, and no reasons for interrupting the study were identified. All experiments were also performed in accordance with relevant guidelines and regulations.

### Setting

This trial was implemented as part of a CMAM^11^ programme in 3 health districts in the central region of Malawi. A health district is divided into sub-administrative areas, 21 of which were selected for the study, with a community-based feeding centre established in each to serve as day-care feeding centres. Participant recruitment into the study began in September 2015 and ended in June 2016. Treatment follow-up ended in August 2016.

### Participant selection

Study participants were selected from all children aged 6–59 months who had been diagnosed with SAM and admitted into the CMAM programme operated by the Ministry of Health. SAM was defined as a MUAC < 115 mm or bilateral pitting oedema of any degree. Children with a MUAC < 115 mm and those with grade 1 or 2 bilateral pitting oedema with good appetite and no medical complications were admitted directly into the day-care programme and enrolled in the study. Re-confirmation of SAM diagnosis was carried out by senior supervisors before enrolment in the study.

This study used simple randomization, with each of the 21 sites recruiting subjects into each of the 3 study arms, at a ratio of 1:1:1. After confirming the subjects’ eligibility for study inclusion, we used a closed envelope method to randomly assign children to receive the FSMS-RUTF, MSMS-RUTF, or PM-RUTF treatment. The trial statistician prepared a computer-generated sequentially numbered randomization list that contained the allocations and codes for each site. These data were sent to the national study coordinator, who then assigned participants to groups at the time of enrolment. The required sample size was achieved in June 2018. More details have been provided elsewhere^13^.

### Subgroup of oedematous and non-oedematous malnutrition

All children were clinically assessed at admission. Children diagnosed with SAM (MUAC < 115 mm) with nutritional bilateral pitting oedema were classified in the oedematous malnutrition (kwashiorkor) subgroup, and those without oedema were classified in the non-oedematous malnutrition (marasmus) subgroup.

### Non-inferiority margin assumption

To date, there are no official clinical values for the concentrations of plasma amino acids in SAM. Therefore, to define a margin for the non-inferiority analysis, we used circadian values of plasma amino acids (oscillating more than 25% during the day) and the difference in plasma amino acid concentrations between healthy infants and those with severe malnutrition (difference of more than 35%). Based on these known oscillation levels, the non-inferiority margin was set at the most conservative value of − 25% of the control (PM-RUTF) as described previously^17^.

### Sample size

Sample size calculations were performed based on data from a study comparing different types of RUTF and looking at changes in plasma amino acid concentrations conducted in the Democratic Republic of Congo (DRC) in 2015^30^. Three amino acids, namely, leucine, cystine and methionine, were used. In the DRC trial, the pooled standard deviations (SDs) in the plasma values were 30.2 µM, 9.5 µM and 3.3 µM for leucine, cystine and methionine, respectively. The alpha was set at 0.05. Based on a non-inferiority margin of − 25%, the limits for leucine, cystine and methionine were 18.34 µM, 8.87 µM and 3.42 µM, respectively, giving sample sizes of 34, 15 and 12 patients per arm, respectively, to provide a power of at least 80%. These sample sizes were calculated using the “TrialSize” (ver. 1.3) package for R^37^.

On the basis of the data from the DRC study^30^, we expected 20% loss to follow-up, giving a total required sample size per arm of 43, 19 and 15 patients for leucine, cystine and methionine, respectively. This study was embedded in another study that required 1299 participants^13^ and applied to children whose blood samples could be collected within 48 h. This resulted in a sample size of 499, exceeding the minimum sample size required for this non-inferiority study.

### Treatment protocol

The treatment was based on the CMAM protocol but applied a day-care approach. Enrolled children attended the site daily from 8 a.m. to 4 p.m. Children were offered 200 kcal/kg/day of one of the study RUTFs (Table S8, S9). Children were defined as recovered discharge when they met the recovery criteria (MUAC ≥ 12.5 cm and no oedema for 14 successive days without acute life-threatening infection) after 3 consecutive months of treatment and defined as non-recovered discharge when they did not meet the recovery criteria after 3 consecutive months of treatment. For the oedematous subgroup, the percentages of children who achieved recovery at discharge were 97.8% (44 of 45), 97% (65 of 67), and 98.2% (55 of 56) for the FSMS-RUTF, MSMS-RUTF, and PM-RUTF arms, respectively. For the non-oedematous subgroup, the percentages were 90.6% (48 of 53), 87.5% (49 of 56), and 92.2% (59 of 64) for the FSMS-RUTF, MSMS-RUTF, and PM-RUTF arms, respectively.

The recovery rates of the FSMS-RUTF and MSMS-RUTF groups were not inferior to the recovery rate of the PM-RUTF group, as described in our previous paper^13^. Further details and the nutritional compositions of the study RUTFs (FSMS, MSMS and PM) are provided in our previous paper^13^.

### Sample collection and amino acid analysis

Blood sampling for amino acid analysis was performed on the subjects from whom blood samples could be collected within 48 h after enrolment, and collections continued until the planned sample number was reached (n = 499). The flow diagram of the participants is indicated in Fig. 2. Blood was sampled at admission and discharge. The sampling procedure was described in a previous paper^17^. The concentrations of amino acid (methionine, leucine, valine, isoleucine, lysine, phenylalanine, tryptophan, threonine, histidine and cystine, which is a cysteine dimer) were measured by an automatic amino acid analyser (L-8800; Hitachi High-Technologies Corporation, Tokyo, Japan). Amino acids were separated by cation-exchange chromatography on a cross-linked sulfonated polystyrene resin column with a modified lithium citrate buffer system^38^. After separation, amino acids were reacted with ninhydrin reagent and detected spectrophotometrically (absorption maximum 570 nm) as derivative forms^39^.

The concentrations of total branch chain amino acids (BCAAs; leucine, valine and isoleucine) and total EAAs (methionine, leucine, valine, isoleucine, lysine, phenylalanine, tryptophan, threonine, histidine) were calculated as the sum of the concentration of each BCAA and each EAA, respectively. Plasma amino acid concentrations are presented as micromolar (μM) units.

### Data analysis

The mean and SD/standard error (SE) or proportion and 95% confidence interval (CI) were used to describe the distributions of the values at admission and discharge as appropriate. The between-group differences in the different parameters are presented with their 95% CIs, and Tukey’s multiple comparisons test or Fisher’s exact test was used for comparisons of baseline characteristics. All statistical analyses were performed using R (ver. 3.5.3)^40^.

Since individual patients were selected from the 21 sites, the data structure is multilevel. Hence, we adopted mixed models for the analysis, which is recommended generally for multilevel data analysis^41,42^. Fixed effects in the model included the intervention (FSMS-RUTF, MSMS-RUTF or PM-RUTF), sampling point (admission or discharge) and their interaction. Because plasma amino acids vary greatly depending on the individual or environment, the model was adjusted for individual admission values and the random effects of CMAM sites. The following generic model was run for amino acid data in each SAM clinical form:

Subject ID: i; Site ID: s; Intervention: v; Sampling point: t; Admission value: b; Amino acid concentration: Y

$$ \begin{gathered} {\text{Y}}_{{{\text{ist}}}} = \, \beta_{{1}} {\text{v}}_{{\text{i}}} + \, \beta_{{2}} {\text{t }} + \, \beta_{{3}} {\text{v}}_{{\text{i}}} *{\text{t }} + \, \beta_{{4}} {\text{b}}_{{\text{i}}} + \, \varepsilon_{{\text{i}}} + {\text{ r}}_{{\text{s}}} \hfill \\ {\text{r}}_{{\text{s}}} \sim N\left( {0, \, \sigma^{{2}} } \right). \hfill \\ \end{gathered} $$

The “admission value” variable included the effect of interindividual differences in plasma amino acid concentrations. To determine the model coefficients, the model was fitted by a residual maximum likelihood estimation with the “lme4” (ver. 1.1.21) package for R^43^. Estimated coefficients (Table S7) were used for the prediction of amino acid concentrations (Fig S2) or statistical tests. The predicted plasma amino acid concentrations at discharge were tested in a non-inferiority hypothesis that those of FSMS-RUTF and MSMS-RUTF would not be less than those of PM-RUTF. For the non-inferiority test, the 2-sided 95% CI of the differences between the FSMS-RUTF arm and the PM-RUTF arm and between the MSMS-RUTF arm and the PM-RUTF arm were estimated by simultaneous inference procedures with the “multcomp” (ver. 1.4.10) package for R^44^. The significance cut-offs for CI estimation were adjusted in each amino acid to avoid a Type 1 error. Bonferroni correction was adopted for the adjustment. Estimated differences are shown as relative values based on the mean plasma amino acid concentration of the PM-RUTF arm at discharge.

To predict the change in the plasma amino acid concentration with treatment according to the two clinical forms of SAM, we applied the following generic model. Fixed effects in the model included SAM clinical forms (oedematous and non-oedematous malnutrition), sampling points (admission or discharge) and their interaction. The model included the random effects of sites. We applied the following generic model for all data sets:

Subject ID: i; site ID: s; SAM clinical form: a; sampling point: t; amino acid concentration: Y

$$ \begin{gathered} {\text{Y}}_{{{\text{ist}}}} = \, \beta_{{1}} {\text{a}}_{{\text{i}}} + \, \beta_{{2}} {\text{t }} + \, \beta_{{3}} {\text{a}}_{{\text{i}}} *{\text{t }} + \, \varepsilon_{{\text{i}}} + {\text{ r}}_{{\text{s}}} \hfill \\ {\text{r}}_{{\text{s}}} \sim N\left( {0, \, \sigma^{{2}} } \right). \hfill \\ \end{gathered} $$

## Supplementary Information

Supplementary Dataset.

Supplementary Information.

## Data Availability

All data analysed during this study are included in this published article (and its Supplementary Information files).
