# Supplementary material for: Amino acid-enriched plant-based RUTF treatment was not inferior to peanut-milk RUTF treatment in restoring plasma amino acid levels among patients with oedematous or non-oedematous malnutrition
Source: Sci Rep. 2021 Jun 15;11:12582. doi: 10.1038/s41598-021-91807-x (PMC8206220; doi:10.1038/s41598-021-91807-x)
Supplement: Supplementary file 2 — Supplementary Information. [file 41598_2021_91807_MOESM2_ESM.docx]

**Amino acid-enriched plant-based RUTF treatment was not inferior to peanut-milk RUTF treatment in restoring plasma amino acid levels among patients with oedematous or non-oedematous malnutrition**

Wataru Sato^1^, Chie Furuta^2^, Peter Akomo^3^, Paluku Bahwere^5,4^, Steve Collins^4,3^, Kate Sadler^4^, Chrissy Banda^4^, Elizabeth Maganga^6^, Sylvester Kathumba^6^, Hitoshi Murakami^2*^

^1^ Ajinomoto Co. Inc., Research Institute for Bioscience Products & Fine Chemicals, Kawasaki-city, Japan

^2^ Ajinomoto Co. Inc., Institute of Food Science and Technologies, Kawasaki-city, Japan

^3^Valid Nutrition, Cork, Ireland

^4^Valid International, Oxford, United Kingdom

^5^Center for Epidemiology, Biostatistics, and Clinical Research, School of Public Health, Free University of Brussels, Brussels, Belgium

^6^Ministry of Health Malawi, Lilongwe, Malawi

*Corresponding Author: Suzukicho 1-1, Kawasaki-ku, Kawasaki, Kanagawa, 210-8681, Japan; E-mail: [hitoshi_murakami@ajinomoto.com](mailto:hitoshi_murakami@ajinomoto.com)

**Supplementary material**

**Table S1**. Testing the non-inferiority of the restoration of plasma amino acid concentrations at discharge between the PM-RUTF arm and the FSMS-RUTF and MSMS-RUTF arms in each SAM subgroup

**Table S2.** Raw plasma amino acid concentrations [μM] in each arm and SAM subgroup

**Table S3.** Changes in raw plasma amino acid concentrations [μM] from admission to discharge in each arm and SAM subgroup.

**Table S4.** Raw plasma amino acid concentrations [μM] in each SAM subgroup

**Table S5.** Changes in raw plasma amino acid concentrations [μM] from admission to discharge in each SAM subgroup.

**Table S6.** Testing the non-inferiority of the recovery rate at discharge between the PM-RUTF arm and the FSMS-RUTF and MSMS-RUTF arms in each SAM subgroup

**Fig S1A.** Raw data of the plasma methionine concentration in each SAM subgroup

**Fig S1B.** Raw data of the plasma leucine concentration in each SAM subgroup

**Fig S1C.** Raw data of the plasma valine concentration in each SAM subgroup

**Fig S1D.** Raw data of the plasma isoleucine concentration in each SAM subgroup

**Fig S1E.** Raw data of the plasma lysine concentration in each SAM subgroup

**Fig S1F.** Raw data of the plasma phenylalanine concentration in each SAM subgroup

**Fig S1G.** Raw data of the plasma tryptophan concentration in each SAM subgroup

**Fig S1H.** Raw data of the plasma threonine concentration in each SAM subgroup

**Fig S1I.** Raw data of the plasma histidine concentration in each SAM subgroup

**Fig S1J.** Raw data of the plasma total BCAA concentration in each SAM subgroup

**Fig S1K.** Raw data of the plasma total EAA concentration in each SAM subgroup

**Fig S1L.** Raw data of the plasma cystine concentration in each SAM subgroup

**Fig S2.** Schematic of the predicted value

**Table S7.** The model coefficients

**Table S8.** Nutritional composition of the study RUTFs

**Table S9.** Comparison of the amino acid profiles of the study RUTFs obtained by laboratory analysis

**Supplementary Data.xlsx** Raw data of the plasma amino acids

**Table S1. Testing non-inferiority of the plasma amino acid concentrations at discharge between the PM-RUTF arm and the FSMS-RUTF and MSMS-RUTF arms in each SAM subgroup^1^**

^1^ The point estimate and 95% CI of the difference in plasma amino acid concentrations at discharge between the FSMS-RUTF and PM-RUTF arms and between the MSMS-RUTF and PM-RUTF arms by using the SAM subgroup data. The differences are shown as actual values based on the plasma amino acid concentration of the PM-RUTF arm at discharge.

^2^ CIs were estimated by simultaneous inference procedures in a mixed model.

^3^ The non-inferiority margin was -25% of the plasma amino acid concentration of the PM-RUTF arm at discharge. Therefore, the lower limit of 95% CI larger than -25% indicated non-inferiority.

**Table S2. Raw plasma amino acid concentrations^1^ [μM] in each arm and SAM subgroup**

^1^Actual plasma amino acid concentration data for subjects with no missing values on either admission or discharge were aggregated. Each amino acid concentration is expressed as the means ± SEs [μM].

**Table S3. Changes in raw plasma amino acid concentrations^1^ [μM] from admission to discharge in each arm and SAM subgroup.**

^1^Actual plasma amino acid concentration data for subjects with no missing values on either admission or discharge were aggregated. Each amino acid concentration is expressed as the means ± SEs [μM].

**Table S4. Raw plasma amino acid concentrations^1^ [μM] in each SAM subgroup**

^1^Actual plasma amino acid concentration data for subjects with no missing values on either admission or discharge were aggregated. Each amino acid concentration is expressed as the means ± SEs [μM].

**Table S5. Changes in raw plasma amino acid concentrations^1^ [μM] from admission to discharge in each SAM subgroup.**

^1^Actual plasma amino acid concentration data for subjects with no missing values on either admission or discharge were aggregated. Each amino acid concentration is expressed as the means ± SEs [μM].

**Table S6.** Testing non-inferiority of the recovery rate at discharge between the PM-RUTF arm and the FSMS-RUTF and MSMS-RUTF arms in each SAM subgroup^1^

^1^ This analysis was performed on the dataset of the original trial (Bahwere et al., AJCN 2017).

^2^ The point estimate and 95% CI of the differences in recovery rate at discharge between the FSMS-RUTF and PM-RUTF arms and between the MSMS-RUTF and PM-RUTF arms by using the SAM subgroup data.

^3^ The non-inferiority margin was -10%. FSMS-RUTF was not inferior to P-RUTF in terms of recovery rate either in the non-oedematous or SAM group in the oedematous group. Similarly, MSMS was not inferior to P-RUTF in terms of recovery rate either in the non-oedematous or SAM group in the oedematous group.

**Fig S1A.** **Raw data of the plasma methionine concentration in each SAM subgroup**

Black circles indicate each subject concentration, and black lines connect the same subject. Blue lines indicate mean concentration.

**Fig S1B. Raw data of the plasma leucine concentration in each SAM subgroup**

Black circles indicate each subject concentration, and black lines connect the same subject. Blue lines indicate mean concentration.

**Fig S1C. Raw data of the plasma valine concentration in each SAM subgroup**

Black circles indicate each subject concentration, and black lines connect the same subject. Blue lines indicate mean concentration.

**Fig S1D. Raw data of the plasma isoleucine concentration in each SAM subgroup**

Black circles indicate each subject concentration, and black lines connect the same subject. Blue lines indicate mean concentration.

**Fig S1E. Raw data of the plasma lysine concentration in each SAM subgroup**

Black circles indicate each subject concentration, and black lines connect the same subject. Blue lines indicate mean concentration.

**Fig S1F. Raw data of the plasma phenylalanine concentration in each SAM subgroup**

Black circles indicate each subject concentration, and black lines connect the same subject. Blue lines indicate mean concentration.

**Fig S1G. Raw data of the plasma tryptophan concentration in each SAM subgroup**

Black circles indicate each subject concentration, and black lines connect the same subject. Blue lines indicate mean concentration.

**Fig S1H. Raw data of the plasma threonine concentration in each SAM subgroup**

Black circles indicate each subject concentration, and black lines connect the same subject. Blue lines indicate mean concentration.

**Fig S1I. Raw data of the plasma histidine concentration in each SAM subgroup**

Black circles indicate each subject concentration, and black lines connect the same subject. Blue lines indicate mean concentration.

**Fig S1J. Raw data of the plasma total BCAA concentration in each SAM subgroup**

Black circles indicate each subject concentration, and black lines connect the same subject. Blue lines indicate mean concentration.

**Fig S1K. Raw data of the plasma total EAA concentration in each SAM subgroup**

Black circles indicate each subject concentration, and black lines connect the same subject. Blue lines indicate mean concentration.

**Fig S1L. Raw data of the plasma cystine concentration in each SAM subgroup**

Black circles indicate each subject concentration, and black lines connect the same subject. Blue lines indicate mean concentration.

**Fig S2.** Schematic of the predicted value

**Table S7.** The model coefficients

**Table S8.** Nutritional composition of the study RUTFs*

^1^FSMS=Milk-free soy-, maize-, and sorghum-based ready-to-use therapeutic food; ^2^MSMS=Milk-, soy-, maize-, and sorghum-based ready-to-use therapeutic food; ^3^PM=Peanut paste-based ready-to-use therapeutic food.

^4^RE, retinol equivalent; TE, total energy; SFs, saturated fatty acids; MUFAs, monounsaturated fatty acids; PUFAs, polyunsaturated fatty acids.

*This table is reused from our primary outcome paper (Bahwere P *et. al.*, AJCN, 2017).

**Table S9.** Comparison of the amino acid profiles of the study RUTFs obtained by laboratory analysis*

^1^FSMS=Milk-free soy-, maize-, and sorghum-based ready-to-use therapeutic food; ^2^MSMS= Milk-, soy-, maize-, and sorghum-based ready-to-use therapeutic food; ^3^PM= Peanut paste-based ready-to-use therapeutic food; ^4^Glu+Gln=Glutamic acid or glutamine; ^5^Asp+Asn=Aspartic acid or asparagine

*This table is reused from our primary outcome paper (Bahwere P *et. al.*, AJCN, 2017).
